# Characterization of a thermotolerant aryl-alcohol oxidase from *Moesziomyces antarcticus* oxidizing 5-hydroxymethyl-2-furancarboxylic acid

**DOI:** 10.1007/s00253-021-11557-8

**Published:** 2021-10-13

**Authors:** Alessa Lappe, Nina Jankowski, Annemie Albrecht, Katja Koschorreck

**Affiliations:** Institute of Biochemistry, Heinrich-Heine University Düsseldorf, Universitätsstraße 1, 40225 Düsseldorf, Germany

**Keywords:** Aryl-alcohol oxidase, *Pichia pastoris (Komagataella phaffii)*, 5-hydroxymethylfurfural (HMF), 5-hydroxymethyl-2-furancarboxylic acid (HMFCA), Bioplastics

## Abstract

**Abstract:**

The development of enzymatic processes for the environmentally friendly production of 2,5-furandicarboxylic acid (FDCA), a renewable precursor for bioplastics, from 5-hydroxymethylfurfural (HMF) has gained increasing attention over the last years. Aryl-alcohol oxidases (AAOs) catalyze the oxidation of HMF to 5-formyl-2-furancarboxylic acid (FFCA) through 2,5-diformylfuran (DFF) and have thus been applied in enzymatic reaction cascades for the production of FDCA. AAOs are flavoproteins that oxidize a broad range of benzylic and aliphatic allylic primary alcohols to the corresponding aldehydes, and in some cases further to acids, while reducing molecular oxygen to hydrogen peroxide. These promising biocatalysts can also be used for the synthesis of flavors, fragrances, and chemical building blocks, but their industrial applicability suffers from low production yield in natural and heterologous hosts. Here we report on heterologous expression of a new aryl-alcohol oxidase, *Ma*AAO, from *Moesziomyces antarcticus* at high yields in the methylotrophic yeast *Pichia pastoris* (recently reclassified as *Komagataella phaffii*)*.* Fed-batch fermentation of recombinant *P. pastoris* yielded around 750 mg of active enzyme per liter of culture. Purified *Ma*AAO was highly stable at pH 2–9 and exhibited high thermal stability with almost 95% residual activity after 48 h at 57.5 °C. *Ma*AAO accepts a broad range of benzylic primary alcohols, aliphatic allylic alcohols, and furan derivatives like HMF as substrates and some oxidation products thereof like piperonal or perillaldehyde serve as building blocks for pharmaceuticals or show health-promoting effects. Besides this, *Ma*AAO oxidized 5-hydroxymethyl-2-furancarboxylic acid (HMFCA) to FFCA, which has not been shown for any other AAO so far. Combining *Ma*AAO with an unspecific peroxygenase oxidizing HMFCA to FFCA in one pot resulted in complete conversion of HMF to FDCA within 144 h. *Ma*AAO is thus a promising biocatalyst for the production of precursors for bioplastics and bioactive compounds.

**Key points:**

• *MaAAO from M. antarcticus was expressed in P. pastoris at 750 mg/l.*

• *MaAAO oxidized 5-hydroxymethyl-2-furancarboxylic acid (HMFCA).*

• *Complete conversion of HMF to 2,5-furandicarboxylic acid by combining MaAAO and UPO.*

**Supplementary Information:**

The online version contains supplementary material available at 10.1007/s00253-021-11557-8.

## Introduction

In times of an emerging importance of a sustainable bioeconomy, biocatalytic processes have gained more and more attention as a promising alternative to chemical synthesis by utilizing enzymes with high activity and product selectivity. Enzymes are able to convert readily available bio-based raw materials under mild reaction conditions into valuable compounds like building blocks for pharmaceuticals, flavors, and fragrances or precursors for polymers (Wiltschi et al. 2020). Among them, aryl-alcohol oxidases (AAOs) have emerged as promising biocatalysts. AAOs (EC 1.1.3.7) are FAD-dependent oxidoreductases that belong to the glucose-methanol-choline (GMC) oxidoreductase superfamily and play an essential role in biomass degradation as they supply peroxide-dependent ligninolytic enzymes with hydrogen peroxide. Although most AAOs described so far have been found in basidiomycetous and ascomycetous fungi, enzymes with AAO activity have been identified in bacteria, insects, and gastropods as well (Ferreira et al. 2015; Serrano et al. 2020; Urlacher and Koschorreck 2021). AAOs typically oxidize benzylic and polyunsaturated aliphatic primary alcohols to the corresponding aldehydes via hydrogen abstraction and transfer to molecular oxygen to produce hydrogen peroxide (Guillen et al. 1992). Hydrated aldehydes (*gem*-diols) can be further oxidized to the corresponding acids, but efficiencies are much lower (Ferreira et al. 2010).

Their broad range of oxidized substrates with the only need of molecular oxygen offers a huge potential of AAOs for biotechnological applications. Besides being used as hydrogen peroxide supplier for peroxide-dependent enzymes in delignification or dye decolorization processes, AAOs can be applied for the production of chemical building blocks, flavors, and fragrances (Serrano et al. 2020; Urlacher and Koschorreck 2021). *Trans*-2-hexenal, used in the flavor and fragrance industry as fresh flavor in foods, was recently produced in a two liquid phase system by selective oxidation of *trans*-2-hexen-1-ol by *P. eryngii* AAO with a turnover number of over 2 million (de Almeida et al. 2019; van Schie et al. 2018). *Pe*AAO2 from *P. eryngii* P34 was shown to oxidize piperonyl alcohol to the fragrance compound piperonal (Jankowski et al. 2020), which is also an important precursor for the synthesis of pharmaceuticals and insecticides (Brum et al. 2019; Santos et al. 2004). The biotechnological potential of AAO was further demonstrated by engineering AAO from *P. eryngii* for selective oxidation of chiral secondary benzyl alcohols (Serrano et al. 2019b; Viña-Gonzalez et al. 2019). This allows for kinetic resolution of racemic secondary alcohols used as building blocks for pharmaceuticals without the need of external cofactors.

**Fig. 1:**
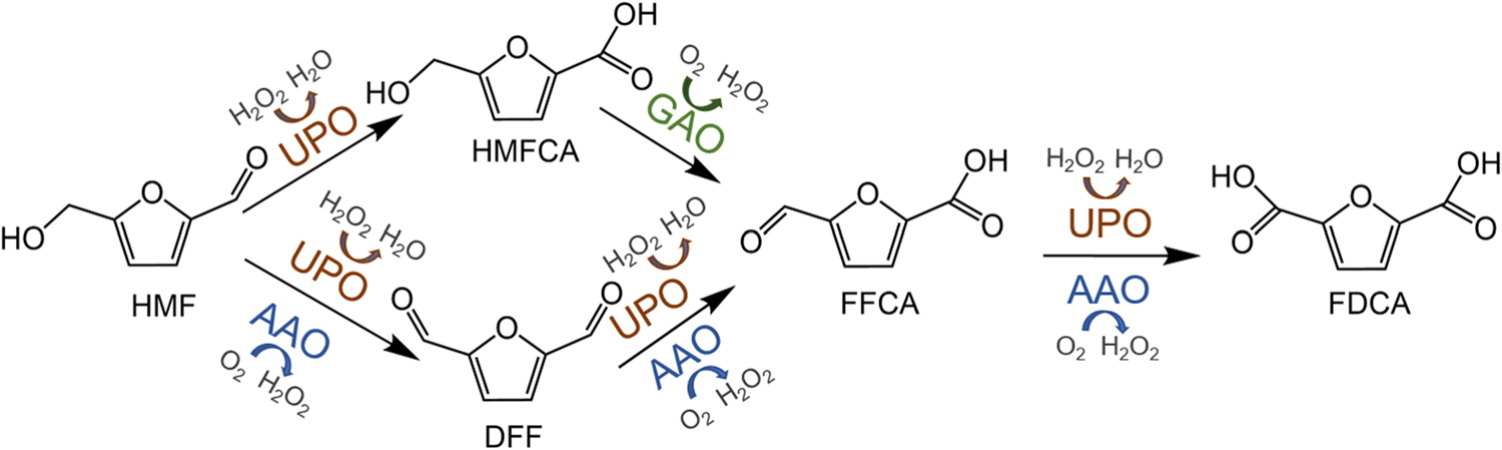
Reaction scheme of HMF oxidation to FDCA employing AAO, UPO, and GAO

Besides this, AAO was applied for the synthesis of 2,5-furandicarboxylic acid (FDCA), a promising renewable building block that is of special interest for the production of bio-based polyesters (polyethylene furanoate (PEF)). FDCA can be produced from 5-hydroxymethylfurfural (HMF) which is obtained from, e.g., cellulose through hydrolysis of cellulose to glucose, followed by acid-mediated isomerization of glucose to fructose, and finally acid-catalyzed dehydration of fructose to HMF (Menegazzo et al. 2018). AAO was shown to oxidize HMF predominantly to 5-formyl-2-furancarboxylic acid (FFCA) via 2,5-diformylfuran (DFF), while oxidation of FFCA to FDCA is rather low and inhibited by hydrogen peroxide formed in course of the reaction (Serrano et al. 2019a). Addition of catalase (Serrano et al. 2019a) or establishment of a three-enzyme system, consisting of AAO, unspecific peroxygenase (UPO), and galactose oxidase (GAO) (Karich et al. 2018), resulted in complete conversion of HMF to FDCA. The latter approach applied H_2_O_2_-dependent UPO to oxidize HMF to 5-hydroxymethyl-2-furancarboxylic acid (HMFCA) and FFCA to FDCA while AAO and GAO provided H_2_O_2_ for UPO by oxidizing HMF to FFCA (catalyzed by AAO) and HMFCA to FFCA (catalyzed by GAO), respectively (Fig. 1). Furthermore, combinatorial saturation mutagenesis was applied to engineer *P. eryngii* AAO for the stepwise oxidation of HMF to FDCA (Vina-Gonzalez et al. 2020). The evolved Bantha variant showed a sixfold improved production of FDCA starting from HMF compared to the wild-type.

However, despite their huge biotechnological potential, only a limited number of AAOs have been described so far and industrial processes applying AAOs have not been established yet which might be due to their difficult expression in natural and heterologous hosts suffering from low yields or requiring tedious in vitro refolding (Ruiz-Duenas et al. 2006; Vina-Gonzalez et al. 2018).

Here, we report on the heterologous expression of a new, thermotolerant AAO, *Ma*AAO from *Moesziomyces antarcticus*, at high yields in *P. pastoris* with promising biocatalytic properties. The enzyme showed a broad activity towards benzylic and polyunsaturated aliphatic primary alcohols as well as furan-derived alcohols and aldehydes which makes this enzyme a promising biocatalyst for the synthesis of bio-based polyesters, fragrances, and bioactive compounds.

## Materials and methods

### Strains, plasmid, and chemicals

Plasmids were propagated in *Escherichia coli* DH5α (Clontech Laboratories Inc., Heidelberg, Germany). *Pichia pastoris* strain X-33 (currently reclassified as *Komagataella phaffii*) was used for heterologous expression of *Ma*AAO and purchased from Invitrogen (Carlsbad, USA). pPICZA_*maaao* was purchased from BioCat GmbH (Heidelberg, Germany). Chemicals and enzymes were purchased from abcr GmbH (Karlsruhe, Germany), Acros Organics (Geel, Belgium), Alfa Aesar (Kandel, Germany), AppliChem GmbH (Darmstadt, Germany), BLDpharm (Shanghai, China), Carl Roth GmbH + Co. KG (Karlsruhe, Germany), Carbolution Chemicals GmbH (St. Ingbert, Germany), Fluorochem (Hadfield, UK), IoLiTec (Heidelberg, Germany), J&K Scientific (Lommel, Belgium), New England Biolabs (Frankfurt am Main, Germany), Sigma-Aldrich (Schnelldorf, Germany), Thermo Fisher Scientific (Bremen, Germany), TCI Chemicals (Eschborn, Germany), and VWR (Darmstadt, Germany).

### Strain construction and expression

The gene encoding for *Ma*AAO from *Moesziomyces antarcticus* (GenBank accession number XM_014798063.1) was codon optimized by JCat (http://www.jcat.de/) for expression in *Saccharomyces cerevisiae* (GenBank accession number MZ574089). The gene was synthesized and ligated into the pPICZA vector by BioCat GmbH (Heidelberg, Germany) using restriction sites BstBI and NotI. Chemically competent *E. coli* DH5α cells were transformed with pPICZA_*maaao* and plasmid isolation was carried out using the ZR Plasmid Miniprep Kit (Zymo Research, Irvine, USA) according to the manufacturer’s instructions. *P. pastoris* X-33 cells were transformed with MssI linearized pPICZA_*maaao* by electroporation. Recombinant cells were selected on yeast extract peptone dextrose sorbitol agar plates (YPDS; 10 g/l yeast extract, 20 g/l peptone, 20 g/l glucose, 1 M sorbitol, 20 g/l agar) supplemented with 100 µg/ml of Zeocin™ (InvivoGen, San Diego, USA). Cells were grown for 4 days at 30 °C. For expression of *Ma*AAO in shaking flasks, several *P. pastoris* transformants were grown in 10 ml buffered complex glycerol medium (BMGY; 10 g/l yeast extract, 20 g/l peptone, 100 mM potassium phosphate buffer pH 6.0, 13.4 g/l yeast nitrogen base without amino acids, 0.4 mg/l biotin, 10 g/l glycerol) at 30 °C and 200 rpm overnight. Precultures were used for inoculation of 100 ml buffered methanol complex medium (BMMY; same as BMGY but with 0.5% methanol instead of glycerol) or 100 ml buffered methanol minimal medium (BMM; 13.4 g/l yeast nitrogen base without amino acids, 100 mM potassium phosphate buffer pH 6.0, 0.4 mg/l biotin, 0.5% (v/v) methanol) to an optical density at 600 nm (OD_600_) of 0.5 and cells were grown for 3 days at 25 °C and 200 rpm. Methanol (0.5% (v/v)) was added daily. Volumetric activity of the cell-free supernatants was measured daily towards veratryl alcohol. The measurements were conducted in 100 mM potassium phosphate buffer pH 6.0 with 5 mM veratryl alcohol at 25 °C. Formation of veratraldehyde (ε_310_ = 9,300 M^−1^ cm^−1^) (Guillen et al. 1992) was followed at 310 nm using an Infinite™ M200 PRO plate reader (Tecan, Männedorf, Switzerland). One unit is defined as the amount of enzyme that converts 1 µmol substrate per minute.

### Fed-batch fermentation and enzyme purification

Fed-batch fermentation of the most active *P. pastoris* transformant was conducted in a 7.5 l bioreactor (Infors, Bottmingen, Switzerland) as described earlier (Jankowski et al. 2020). Samples were taken daily to monitor OD_600_, volumetric activity towards veratryl alcohol, and protein concentration. After 8 days of cultivation, the fermentation broth was harvested by centrifugation for 15 min at 10,000 g and 4 °C. The cell-free supernatant was concentrated and rebuffered in 50 mM potassium phosphate buffer pH 6.0 by tangential flow filtration with cut-off membranes of 10 kDa (Pall, Port Washington, USA). The concentrated supernatant was supplemented with ammonium sulfate to a final concentration of 1.5 M. The sample was centrifuged for 30 min at 18,000* g* and 4 °C and filtered using a 0.45 µm pore size filter. Five milliliters of sample was loaded onto a Butyl Sepharose HP column (GE Healthcare, Chicago, USA) on an ÄKTApurifier FPLC-system (GE Healthcare). The column was washed with two column volumes (CVs) of 50 mM potassium phosphate buffer pH 6.0 with 1.5 M ammonium sulfate and proteins were eluted with a linear gradient over 6 CVs to 100% 50 mM potassium phosphate buffer pH 6.0. Active fractions towards veratryl alcohol were pooled, concentrated, and desalted using a Vivaspin Turbo 15 ultrafiltration unit with 10 kDa cut-off (Sartorius, Göttingen, Germany). The concentrated sample was loaded onto a Superdex 200 Increase column (GE Healthcare). Proteins were eluted with one CV of 50 mM potassium phosphate buffer pH 6.0 with 150 mM sodium chloride and active fractions were pooled, concentrated, and desalted as described above. The concentrated sample was loaded onto a DEAE Sepharose FF column (GE Healthcare) equilibrated with 100 mM Tris/HCl buffer pH 8.5. Proteins were eluted with a linear gradient over 3 CVs to 50% of 100 mM Tris/HCl buffer pH 8.5 with 1 M sodium chloride. Active fractions were pooled, concentrated, and rebuffered in 50 mM potassium phosphate buffer pH 6.0 as described above. Purified *Ma*AAO was stored at 4 °C.

### Biochemical characterization

Protein concentration was determined with the Bradford assay (Bradford 1976) with bovine serum albumin (BSA) as standard. Deglycosylation of *Ma*AAO was conducted by treatment with PNGase F (New England Biolabs, Frankfurt am Main, Germany) according to the manufacturer’s instructions. SDS-PAGE analysis was performed according to the protocol of Laemmli (1970). Spectroscopic analysis of *Ma*AAO was performed using a Lambda 35 spectrophotometer (Perkin Elmer, Waltham, USA). The molar extinction coefficient of purified *Ma*AAO was calculated by heat denaturation using ε_450_ = 11,300 M^−1^ cm^−1^ for the free FAD (Aliverti et al. 1999). For determination of the *T*_50_ value (temperature at which the enzyme loses 50% of its activity after heat incubation), purified *Ma*AAO was incubated at temperatures of 30 to 80 °C for 10 min. After cooling on ice, the residual activity towards veratryl alcohol was measured as described above. The *T*_50_ value was estimated by fitting the data to the Boltzmann equation.

### Influence of pH, temperature, hydrogen peroxide, and cosolvents

The influence of pH on activity of *Ma*AAO towards veratryl alcohol, cinnamyl alcohol, and *trans,trans*-2,4-hexadien-1-ol (at a concentration of 5 mM each) was investigated in 100 mM Britton-Robinson buffer pH 2 to 9 at room temperature. Product formation was followed spectrophotometrically using an Infinite™ M200 PRO plate reader. Enzyme activity was calculated by using the molar extinction coefficient of veratraldehyde (ε_310_ = 9,300 M^−1^ cm^−1^) (Guillen et al. 1992), cinnamaldehyde (ε_310_ = 15,600 M^−1^ cm^−1^) (Ferreira et al. 2005), and *trans,trans*-2,4-hexadienal (ε_280_ = 30,140 M^−1^ cm^−1^) (Ruiz-Duenas et al. 2006). pH stability of *Ma*AAO was determined by incubating the purified enzyme in 100 mM Britton-Robinson buffer pH 2 to 9 at room temperature for 48 h. Thermal stability of *Ma*AAO was determined by incubating the purified enzyme at temperatures of 30 to 80 °C in 50 mM potassium phosphate buffer pH 6.0 for 48 h. Samples were taken at certain time points and residual activity towards veratryl alcohol was measured as described above. The influence of increasing concentrations of hydrogen peroxide (H_2_O_2_) on the activity of *Ma*AAO was investigated by measuring the activity towards veratryl alcohol in the presence of 0 to 500 mM H_2_O_2_. Stability towards H_2_O_2_ was determined by incubating the enzyme in 50 mM potassium phosphate buffer pH 6.0 with 0 to 500 mM H_2_O_2_ for up to 48 h. Samples were taken at certain time points and residual activity towards veratryl alcohol was measured as described above. Activity of *Ma*AAO in the presence of up to 40% of dimethyl sulfoxide (DMSO), 2-methyltetrahydrofuran (MeTHF), choline acetate, and choline dihydrogen phosphate towards veratryl alcohol was determined as described above. Stability of *Ma*AAO towards up to 20% of dimethyl sulfoxide (DMSO), 40% of choline acetate, and 40% of choline dihydrogen phosphate was determined by incubating *Ma*AAO in 50 mM potassium phosphate buffer pH 7.5 with the respective cosolvent for 24 h at 25 °C. Residual activity towards veratryl alcohol was measured as described above. All measurements were done in triplicate.

### Substrate screening

Activity of *Ma*AAO towards benzyl alcohol, 4-hydroxybenzyl alcohol, *m*-anisyl alcohol, *p*-anisyl alcohol, veratryl alcohol, isovanillyl alcohol, vanillyl alcohol, 2,4-dimethoxybenzyl alcohol, 3-aminobenzyl alcohol, 4-aminobenzyl alcohol, cumic alcohol, piperonyl alcohol, 1-phenylethanol, 2-naphthalenemethanol, 1-pyrenemethanol, cinnamyl alcohol, coniferyl alcohol, sinapyl alcohol, furfuryl alcohol, furfural, 2,5-diformylfuran (DFF), 5-hydroxymethylfurfural (HMF), 5-hydroxymethyl-2-furancarboxylic acid (HMFCA), 5-formyl-2-furancarboxylic acid (FFCA), prenol, *trans,trans*-2,4-hexadien-1-ol, *trans,trans*-2,4-heptadien-1-ol, 2-thiophenemethanol, 2-pyridinemethanol, (*S*)-perillyl alcohol, eugenol, benzaldehyde, and vanillin was determined by using a coupled assay for detection of H_2_O_2_ generated in course of substrate oxidation via horseradish peroxidase- (HRP, Type VI, Sigma-Aldrich, Schnelldorf, Germany) catalyzed H_2_O_2_-dependent oxidation of 2,6-dimethoxyphenol (2,6-DMP) to coerulignone. Stock solutions of substrates were prepared in 100 mM potassium phosphate buffer pH 6.0 and dimethyl sulfoxide, respectively, at a concentration of 50 and 500 mM, respectively. The measurements were conducted in 100 mM potassium phosphate buffer pH 6.0 with 5 mM substrate, 5 mM 2,6-DMP, 100 µg/ml HRP, and 0.02 µM of purified *Ma*AAO in a total volume of 200 µl at 25 °C. Formation of coerulignone (ε_468_ = 49,600 M^−1^ cm^−1^) was followed at 468 nm using an Infinite™ M200 PRO plate reader. All measurements were conducted in triplicates.

### Determination of kinetic constants

V_max_ and *K*_M_ values were determined for selected substrates in 100 mM potassium phosphate buffer pH 6.0 at 25 °C using an Infinite™ M200 PRO plate reader. Substrate concentrations ranged from 1 µM up to 10 mM (dependent on the substrate). All measurements were done in triplicate. Product formation was followed at 314 nm for *m*-anisaldehyde (ε_314_ = 2,540 M^−1^ cm^−1^) (Guillen et al. 1992), at 285 nm for *p*-anisaldehyde (ε_285_ = 16,980 M^−1^ cm^−1^) (Guillen et al. 1992), at 250 nm for benzaldehyde (ε_250_ = 13,800 M^−1^ cm^−1^) (Guillen et al. 1992), at 310 nm for cinnamaldehyde (ε_310_ = 15,600 M^−1^ cm^−1^) (Ferreira et al. 2005), at 314 nm for 2,4-dimethoxy benzaldehyde (ε_314_ = 8,840 M^−1^ cm^−1^) (Guillen et al. 1992), at 280 nm for *trans,trans*-2,4-hexadienal (ε_280_ = 30,140 M^−1^ cm^−1^) (Ruiz-Duenas et al. 2006), at 307 nm for isovanillin (ε_307_ = 7,383 M^−1^ cm^−1^) (Ferreira et al. 2005), at 317 nm for piperonal (ε_317_ = 8,680 M^−1^ cm^−1^) (Jankowski et al. 2020), and at 309 nm for vanillin (ε_309_ = 8,332 M^−1^ cm^−1^) (Ferreira et al. 2005). For 3-aminobenzyl alcohol, HMF and (*S*)-perillyl alcohol the coupled 2,6-DMP-HRP assay was applied for determination of kinetic constants as described above. Data were fitted to the Michaelis–Menten equation or substrate excess inhibition equation (v = V_max_*[S]/(*K*_M_ + [S]*(1 + [S]/*K*_i_)) using OriginPro 9.0. *k*_cat_ values were calculated based on the molar concentration of *Ma*AAO determined by using the molar extinction coefficient.

### Oxidation of HMF and its oxidized derivatives

HMF, DFF, HMFCA, and FFCA, respectively, were incubated at a concentration of 2 mM in 100 mM sodium acetate buffer pH 5.0 and 100 mM sodium phosphate buffer pH 6.0, respectively, with 2 µM *Ma*AAO in a total volume of 200 µl at 25 °C under shaking conditions for up to 6 days. Samples were taken in course of reaction. Reactions were stopped by adding 10 µl 6 M HCl. 2-furoic acid was added as internal standard at a final concentration of 2 mM. Samples were extracted two times with 200 µl methyl *tert*-butyl ether (MTBE), dried over MgSO_4_, evaporated, resuspended in *N,O*-bis(trimethylsilyl)trifluoroacetamide (BSTFA) for derivatization, and incubated for 15 min at 30 °C prior to GCMS analysis.

The two-enzyme setup for HMF conversion consisted of 2 mM HMF in 100 mM sodium phosphate buffer pH 6.0 with 2 μM *Ma*AAO and 2 μM UPO (see supplemental material for description of enzyme preparation) in a total volume of 200 μl. The reaction was shaken at 25 °C for up to 6 days and analyzed as described above.

### GCMS analysis

Oxidation of HMF and its oxidized derivatives was analyzed on a GC–MS-QP-2010 Plus (Shimadzu, Tokyo, Japan) equipped with a FS-Supreme-5 ms column (CS Chromatographie Service GmbH, Langerwehe, Germany) and helium as carrier gas. The injection temperature was 250 °C, the interface was set to 285 °C, and the ion source was set to 200 °C. The column temperature was set to 110 °C, kept for 2 min at this temperature, and ramped to 300 °C at a rate of 20 °C/min. Compounds were identified by comparing the acquired mass spectra with authentic samples or with the NIST 08 database.

## Results

### Heterologous expression of *Ma*AAO

The gene encoding the putative AAO from *M. antarcticus* (XP_014653549.1), annotated as GMC oxidoreductase, was integrated into the genome of *P. pastoris* X-33 (reclassified as *Komagataella phaffii*) under control of the methanol inducible P_*AOX1*_ promoter. The putative AAO was designated *Ma*AAO. Nine *P. pastoris* transformants were screened for secretion of *Ma*AAO in BMMY and BMM medium in shaking flasks using veratryl alcohol as substrate. The best performing transformant yielded a volumetric activity towards veratryl alcohol of 150 U/l after 2 days of expression in BMM medium and was subsequently used for enzyme production in a 3 l fed-batch fermentation process. After 8 days of fed-batch cultivation, the volumetric activity reached 19,200 U/l at an OD_600_ of 394.

### Structural and spectroscopic properties of *Ma*AAO

Purified *Ma*AAO showed a specific activity towards veratryl alcohol of 25.7 U/mg which gave a calculated expression yield of 750 mg/l after 8 days of fed-batch fermentation. *Ma*AAO runs as a single band on SDS-PAGE with a molecular mass of around 75 kDa (Figure S1). After *N*-deglycosylation with PNGase F, the band shifted to around 67 kDa, the calculated theoretical molecular mass of *Ma*AAO which corresponds to 11% of *N*-glycosylation.

**Fig. 2:**
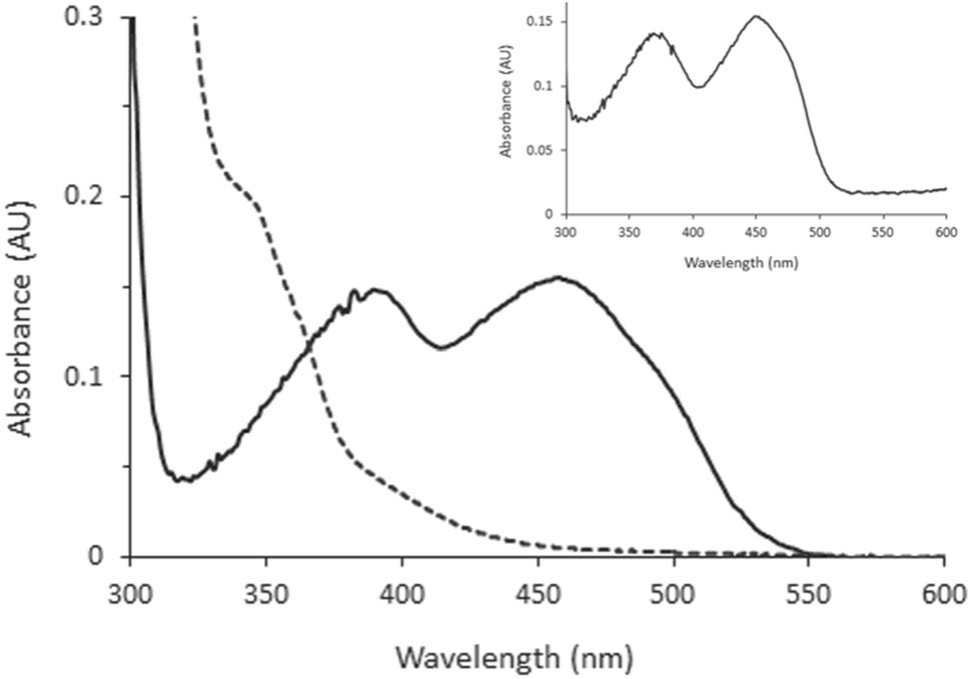
Absorbance spectrum of purified *Ma*AAO. Solid line: oxidized form; dashed line: reduced form after reduction with 1 mM *p*-anisyl alcohol. The inset shows the UV/Vis spectrum of FAD extracted from *Ma*AAO after heat denaturation

Spectroscopic analysis of the yellow enzyme solution revealed two absorbance maxima at 389 nm and 458 nm, typical for flavoproteins (Fig. 2). Upon heat denaturation, an FAD spectrum with absorbance maxima at 376 nm and 450 nm was obtained. The estimated molar extinction coefficient of purified *Ma*AAO at 458 nm was 8,556 M^−1^ cm^−1^.

### Influence of pH, temperature, H_2_O_2_, and cosolvents on enzyme activity and stability

**Fig. 3:**
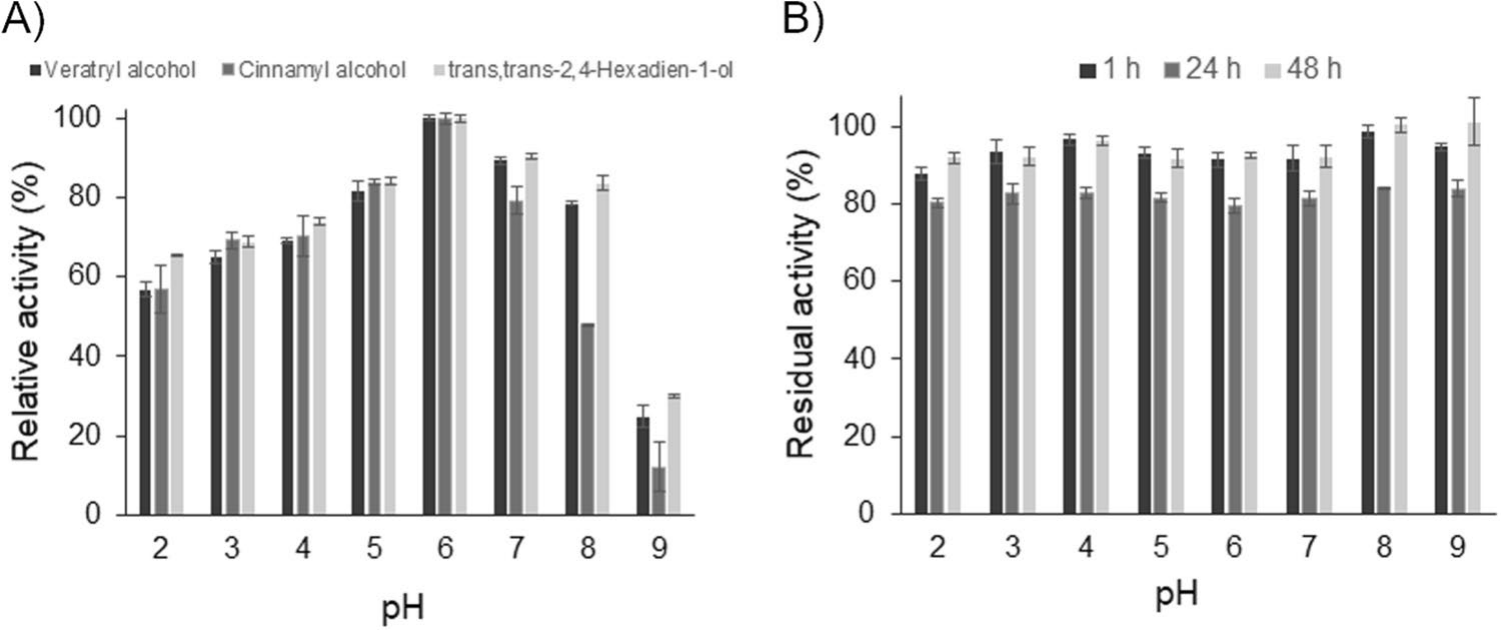
Influence of pH on activity and stability of *Ma*AAO. **A** pH optimum of *Ma*AAO using veratryl alcohol (black bar), cinnamyl alcohol (dark gray bar), and *trans,trans*-2,4-hexadien-1-ol (light gray bar) as substrates determined in 100 mM Britton-Robinson buffer at pH 2–9. Activity at pH 6.0 was set to 100%. **B** pH stability of *Ma*AAO measured after incubation for 1 h (black bar), 24 h (dark gray bar), and 48 h (light gray bar) in 100 mM Britton-Robinson buffer at the corresponding pH value at 25 °C. Initial activity without incubation was set to 100%

**Fig. 4:**
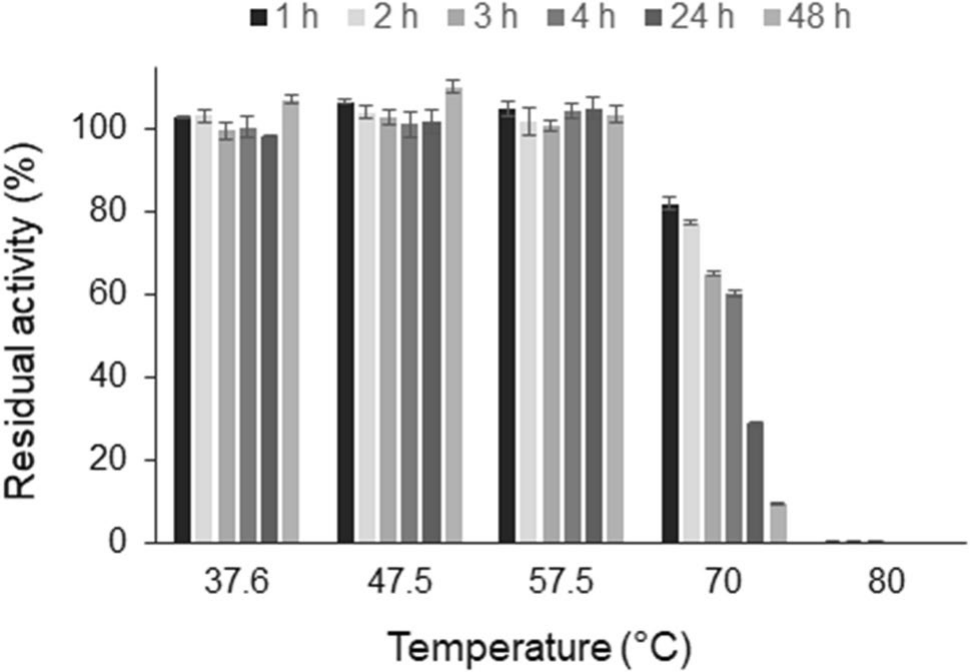
Thermal stability of *Ma*AAO. Residual activity of *Ma*AAO after different times of incubation at 37.6 °C, 47.5 °C, 57.5 °C, 70 °C, and 80 °C in 50 mM potassium phosphate buffer pH 6.0. Initial activity without incubation was set to 100%

Activity of *Ma*AAO towards veratryl alcohol, cinnamyl alcohol, and *trans,trans*-2,4-hexadien-1-ol at pH 2 to 9 was determined. All substrates were oxidized by *Ma*AAO at the investigated pH values. pH optimum of *Ma*AAO for all substrates was at pH 6.0 (Fig. 3A). Stability of *Ma*AAO at pH values of 2 to 9 was investigated. *Ma*AAO retained around 85% of its initial activity after 48 h incubation under neutral, basic, and even acidic conditions (Fig. 3B). Thermal stability of *Ma*AAO was investigated by incubating the enzyme between 30 and 80 °C at pH 6.0 for up to 48 h. *Ma*AAO was stable up to 57.5 °C with almost 95% residual activity after 48 h (Fig. 4). At 70 °C, a residual activity of 60% was found after 3 h of incubation and after 24 h residual activity dropped to 33%. The *T*_50_ value of *Ma*AAO (the temperature at which half of the enzyme activity is lost after 10 min of incubation) was 74 °C.

**Fig. 5:**
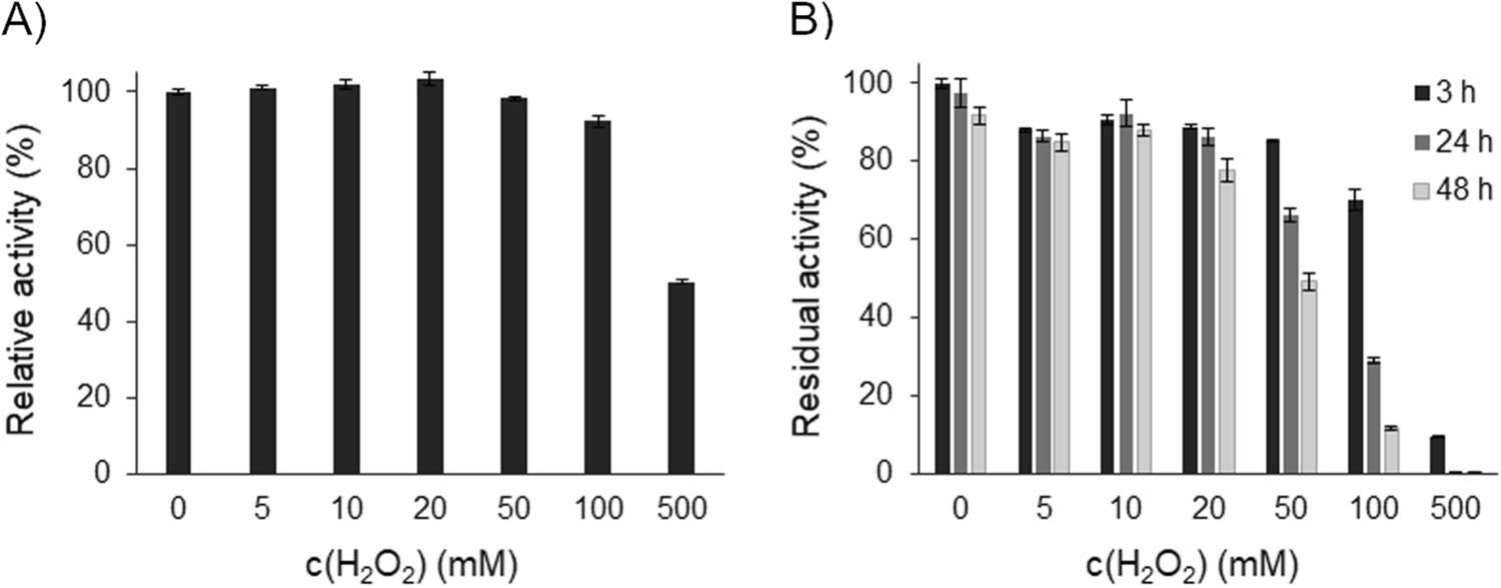
Influence of hydrogen peroxide on activity and stability of *Ma*AAO. **A** Activity of *Ma*AAO towards veratryl alcohol in the presence of 0–500 mM hydrogen peroxide. Relative activity is given in % of enzyme activity without addition of hydrogen peroxide. **B** Residual activity of *Ma*AAO after 3 h (black bar), 24 h (dark gray bar), and 48 h (light gray bar) of incubation with 0-500 mM hydrogen peroxide in 50 mM potassium phosphate buffer pH 6.0 at 25 °C. Initial activity without incubation was set to 100%

The influence of increasing concentrations of H_2_O_2_ on activity and stability of *Ma*AAO was investigated (Fig. 5). Up to 100 mM H_2_O_2_ had a marginal effect on activity of *Ma*AAO (90% of initial activity), but 500 mM H_2_O_2_ resulted in 50% decrease in initial activity. Stability of *Ma*AAO was not affected by incubation with 5 or 10 mM H_2_O_2_ for 48 h (around 90% residual activity) but residual activity dropped to 53% and 13%, respectively, after 48 h incubation with 50 and 100 mM H_2_O_2_, while at 500 mM H_2_O_2_ only 10% residual activity was left after 3 h of incubation.

**Fig. 6:**
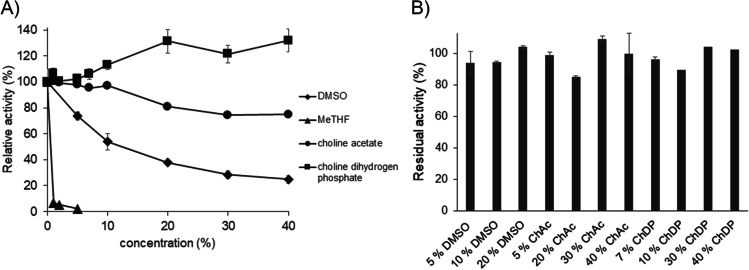
Influence of cosolvents on activity and stability of *Ma*AAO. **A** Activity of *Ma*AAO towards veratryl alcohol in the presence of 0–40% of DMSO (diamonds), MeTHF (triangles), choline acetate (circles), and choline dihydrogen phosphate (squares). Relative activity is given in % of enzyme activity without cosolvents. **B** Residual activity of *Ma*AAO after 24 h incubation with DMSO, choline acetate (ChAc), and choline dihydrogen phosphate (ChDP), respectively, at different concentrations in 50 mM potassium phosphate buffer pH 7.5 at 25 °C. Activity was measured with veratryl alcohol as substrate under standard assay conditions. Initial activity without incubation was set to 100%

Activity and stability of *Ma*AAO in presence of two organic solvents (DMSO and 2-methyltetrahydrofuran (MeTHF)) and two ionic liquids (choline acetate and choline dihydrogen phosphate) were determined. Activity of *Ma*AAO was reduced in the presence of DMSO (Fig. 6A). At 10% DMSO activity dropped to 54% and at 40% DMSO only 24% of its initial activity remained. MeTHF had a more severe effect with 7% of remaining activity at 1% of solvent. Choline acetate and choline dihydrogen phosphate (up to 10%) hardly influenced activity of *Ma*AAO. At 40% choline acetate 74% activity remained while with choline dihydrogen phosphate activity of *Ma*AAO increased to 132% at 40% of the cosolvent. On the other hand, *Ma*AAO was quite stable in presence of DMSO (up to 20%), choline acetate, and choline dihydrogen phosphate (up to 40%) with over 80% remaining activity after 24 h of incubation (Fig. 6B).

### Substrate spectrum of *Ma*AAO

A broad range of primary alcohols and some aldehydes were tested as substrates for *Ma*AAO. For this purpose, a coupled colorimetric assay using 2,6-DMP and horseradish peroxidase to follow hydrogen peroxide production in course of substrate oxidation by *Ma*AAO was applied. The activity towards benzyl alcohol was set to 100%. All benzylic alcohols tested were accepted as substrates with relative activities of up to 250% for veratryl alcohol except for 1-phenylethanol which was not oxidized at all (Table 1). Benzylic alcohols with a methoxy- or amino-substituent at the *meta*- or *para*-position were oxidized equally well (similar relative activity of vanillyl and isovanillyl alcohol and of 3- and 4-amino benzyl alcohol), except for *m*- and *p*-anisyl alcohol with 139% and 219% relative activity, respectively. An extended unsaturated side chain as in cinnamyl alcohol increased activity to 231% as compared to benzyl alcohol, while for coniferyl alcohol activity dropped to 23% and sinapyl alcohol was not oxidized at all. *Ma*AAO showed the highest relative activity of 282% towards the aliphatic alcohol *trans,trans*-2,4-hexadien-1-ol followed by piperonyl alcohol (252%), a benzodioxol derivative. Other tested benzylic alcohols were oxidized as well but with lower activity compared to benzyl alcohol. All furan derivatives tested were oxidized by *Ma*AAO with HMF leading to the highest relative activity of 176%. HMFCA was oxidized by *Ma*AAO with a relative activity of 20%. *Ma*AAO showed a high activity towards (*S*)-perillyl alcohol (185%) while eugenol, a typical substrate of vanillyl alcohol oxidases, was hardly converted (5% relative activity). Aldehydes were oxidized to a much lesser extent than the corresponding alcohols (e.g., 7% relative activity with vanillin compared to 212% with vanillyl alcohol). No activity towards GMC oxidoreductase substrates such as D-glucose, D-galactose, maltose, lactose, methanol, or ethanol was found.

**Table 1:**
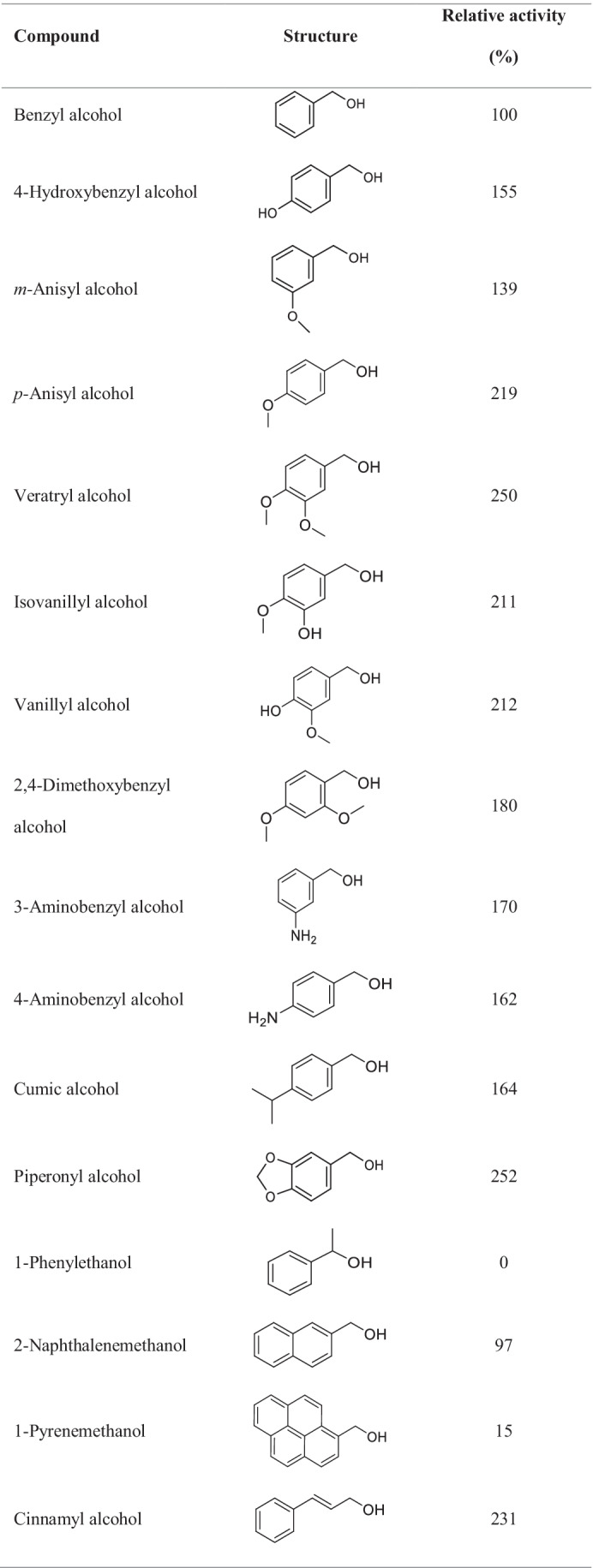

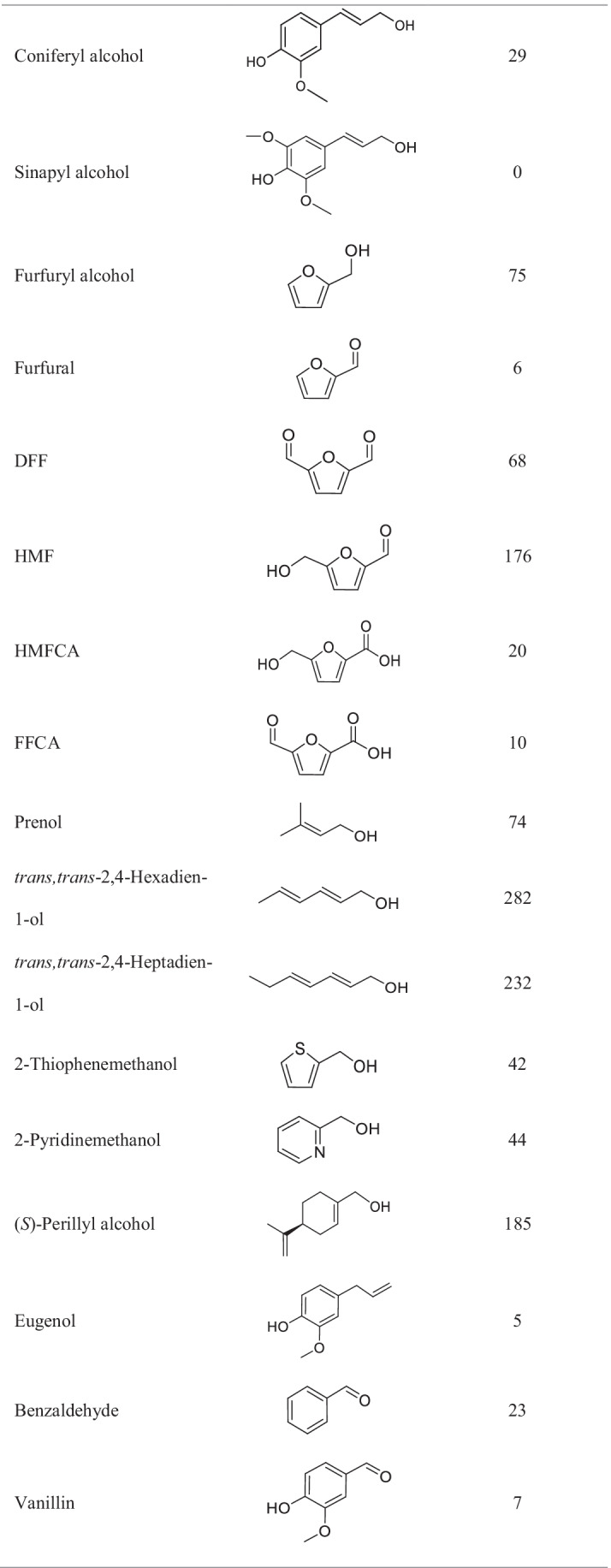
Substrate spectrum of *Ma*AAO. Hydrogen peroxide formed in course of substrate oxidation was detected in a coupled 2,6-DMP-HRP assay. Substrates were used at 5 mM final concentration in 100 mM potassium phosphate buffer pH 6.0. Activity towards benzyl alcohol was set to 100%

For some of the tested substrates, *K*_M_ and *k*_cat_ values of *Ma*AAO were determined (Table 2). While *k*_cat_ values were in the same range for all tested substrates, *K*_M_ values ranged from 1.74 µM for 3-aminobenzyl alcohol to 582 µM for 2,4-dimethoxybenzyl alcohol. Highest affinities and catalytic activities were found for 3-aminobenzyl alcohol, *m*-anisyl alcohol, and *p-*anisyl alcohol. For substrates with *K*_M_ values below 15 µM like 3-aminobenzyl alcohol, *p*- and *m*-anisyl alcohol, and benzyl alcohol, a strong decrease in enzymatic activity was observed at increased substrate concentrations. The data fitted well to the Michaelis–Menten equation derived for excess-substrate inhibition (Figure S2). The calculated *K*_IU_ values are app. 500 times higher than the corresponding *K*_M_ values (Table S1). Substrates with *K*_M_ values between 15 and 100 µM showed moderate inhibition, except for cinnamyl alcohol (no inhibition observed), and no inhibition was detected for substrates with *K*_M_ values above 100 µM. The catalytic efficiency of *Ma*AAO ranged from 15.7 mM^−1^ s^−1^ for HMF to 3670 mM^−1^ s^−1^ for monosubstituted benzylic alcohols. The catalytic efficiency of *Ma*AAO for the non-aromatic (*S*)-perillyl alcohol (387 mM^−1^ s^−1^) was similar to the aromatic vanillyl alcohol (354 mM^−1^ s^−1^).

**Table 2:** Kinetic constants of *Ma*AAO and of other AAOs

		*Ma*AAO from *M. antarcticus*^*a*^	*Pe*AAO2 from *P. eryngii* P34^b^	*Pe*AAO from *P. erygnii*^c^	r*Cc*AAO from *C. cinerea*^d^	BAO from *B. cinerea*^e^	*Um*AAO from *U. maydis*^f^
3-Aminobenzyl alcohol^g^	*K*_M_ (µM)	1.74 ± 0.24	n.d	n.d	n.d	n.d	n.d
	*k*_cat_ (s^−1^)	6.4	n.d	n.d	n.d	n.d	n.d
	*k*_cat_/*K*_M_ (mM^−1^ s^−1^)	3670	n.d	n.d	n.d	n.d	n.d
*m*-Anisyl alcohol	*K*_M_ (µM)	4.43 ± 2.65	n.d	227	3.96 ± 1.14	156 ± 5	n.d
	*k*_cat_ (s^−1^)	12.2	n.d	15	7.66	54	n.d
	*k*_cat_/*K*_M_ (mM^−1^ s^−1^)	2754	n.d	65	1940	349	n.d
*p*-Anisyl alcohol	*K*_M_ (µM)	3.54 ± 0.66	24.3 ± 0.8	27	11.6 ± 1.0	187 ± 16	4.8 ± 0.4
	*k*_cat_ (s^−1^)	10.2	59.2	142	12.5	121	45
	*k*_cat_/*K*_M_ (mM^−1^ s^−1^)	2869	2436	5233	1080	646	9380
Benzyl alcohol	*K*_M_ (µM)	< 15.0	599.6 ± 18.7	632	1.21 ± 0.27	329 ± 15	n.d
	*k*_cat_ (s^−1^)	11.2	12.8	30	6.13	6	n.d
	*k*_cat_/*K*_M_ (mM^−1^ s^−1^)	745	21.39	47	5060	18	n.d
Cinnamyl alcohol	*K*_M_ (µM)	26.9	2740 ± 103	708	n.d	73 ± 3	35 ± 2
	*k*_cat_ (s^−1^)	8.9	125.5	65	n.d	22	88
	*k*_cat_/*K*_M_ (mM^−1^ s^−1^)	332	45.80	78	n.d	305	2510
2,4-Dimethoxybenzyl alcohol	*K*_M_ (µM)	582	n.d	n.d	n.d	n.d	1820 ± 150
	*k*_cat_ (s^−1^)	5.9	n.d	n.d	n.d	n.d	30
	*k*_cat_/*K*_M_ (mM^−1^ s^−1^)	35.2	n.d	n.d	n.d	n.d	16.5
*trans,trans*-2,4-Hexadien-1-ol	*K*_M_ (µM)	26.5 ± 1.7	143.6 ± 11.5	94	15.6 ± 0.8	521 ± 27	15 ± 1
	*k*_cat_ (s^−1^)	11.5	68.8 ± 0.05	119	48.3	97	64
	*k*_cat_/*K*_M_ (mM^−1^ s^−1^)	435	479.3	1271	3100	186	4270
HMF^g^	*K*_M_ (µM)	341 ± 20	n.d	1600 ± 200^ h^	n.d	n.d	n.d
	*k*_cat_ (s^−1^)	5.4	n.d	0.67^ h^	n.d	n.d	n.d
	*k*_cat_/*K*_M_ (mM^−1^ s^−1^)	15.7	n.d	0.42^ h^	n.d	n.d	n.d
Isovanillyl alcohol	*K*_M_ (µM)	60.9 ± 6.7	n.d	831	42 ± 0.9	1115 ± 35	n.d
	*k*_cat_ (s^−1^)	9.6	n.d	127	7.02	56	n.d
	*k*_cat_/*K*_M_ (mM^−1^ s^−1^)	158	n.d	152	167	51	n.d
(*S*)-Perillyl alcohol^g^	*K*_M_ (µM)	23.7 ± 1.7	n.d	n.d	n.d	n.d	n.d
	*k*_cat_ (s^−1^)	9.2	n.d	n.d	n.d	n.d	n.d
	*k*_cat_/*K*_M_ (mM^−1^ s^−1^)	387	n.d	n.d	n.d	n.d	n.d
Piperonyl alcohol	*K*_M_ (µM)	12.2 ± 1.0	59.1 ± 3.0	n.d	n.d	n.d	n.d
	*k*_cat_ (s^−1^)	11.3	35.5	n.d	n.d	n.d	n.d
	*k*_cat_/*K*_M_ (mM^−1^ s^−1^)	926	600.2	n.d	n.d	n.d	n.d
Vanillyl alcohol	*K*_M_ (µM)	30.0 ± 2.4	n.d	n.d	6.27 ± 0.43	1404.0 ± 77	n.d
	*k*_cat_ (s^−1^)	10.6	n.d	n.d	14.7	44	n.d
	*k*_cat_/*K*_M_ (mM^−1^ s^−1^)	354	n.d	n.d	2350	31	n.d
Veratryl alcohol	*K*_M_ (µM)	119.0 ± 7.0	446.6 ± 7.5	540	48.3 ± 6.1	2094 ± 114	120 ± 10
	*k*_cat_ (s^−1^)	11.7	47.2	114	13.2	47	53
	*k*_cat_/*K*_M_ (mM^−1^ s^−1^)	98	105.7	210	273	22	440

*n.d.* not determined

^a^This study, 100 mM sodium phosphate buffer pH 6.0, 25 °C

^b^Jankowski et al. (2020), 100 mM sodium phosphate buffer pH 6.0, 25 °C

^c^Ferreira et al. (2006), 100 mM sodium phosphate buffer pH 6.0, 24 °C

^d^Tamaru et al. (2018), 50 mM potassium phosphate buffer pH 7.0, 25 °C

^e^Goetghebeur et al. (1992), 100 mM sodium phosphate buffer, pH 6.0, 24 °C

^f^Couturier et al. (2016), measured with coupled ABTS-HRP assay in 100 mM McIlvaine buffer pH 6.0, at 30 °C

^g^Measured with coupled 2,6-DMP-HRP assay in 50 mM potassium phosphate buffer pH 6.0, at 25 °C

^h^Values taken from Vina-Gonzalez et al. (2020)

### Oxidation of HMF and its derivatives

Conversion of HMF and its oxidized derivatives DFF, HMFCA, and FFCA by *Ma*AAO was conducted at pH 5 and 6 for 6 days. HMF and DFF were oxidized equally well at both pH values and complete conversion to FFCA was reached after 24 h (Table 3). However, only minor amounts of FDCA with 1% or below were detected even after 6 days of reaction. HMFCA was oxidized best at pH 5 by *Ma*AAO with 60% conversion to FFCA within 24 h, while at pH 6.0 only 25% FFCA was formed after 24 h (data not shown). After 6 days of reaction, full conversion of HMFCA to FFCA was observed. Again, only marginal amounts of FDCA with less than 1% were detected. Oxidation of FFCA by *Ma*AAO was lowest among all tested furan derivatives. After 24 h, only 1% FDCA was detected at all while after 6 days of reaction at pH 6.0 40% FDCA was formed. At pH 5, no oxidation products were detected. Due to the high activity of *Ma*AAO towards HMFCA, a two-enzyme approach consisting of *Ma*AAO and an unspecific peroxygenase was applied for HMF oxidation. With this setup complete conversion of HMF to FDCA was obtained within 6 days.

**Table 3:** Molar percentages after treatment of HMF, DFF, HMFCA, and FFCA, respectively, with *Ma*AAO for 24 h and 144 h. Reactions were performed with 2 mM substrate and 2 µM *Ma*AAO in 100 mM sodium phosphate buffer pH 6.0. HMF was additionally treated with 2 µM *Ma*AAO and 2 µM UPO

Substrate	Enzyme	Time (h)	Molar percentages (%)				
			HMF	DFF	HMFCA	FFCA	FDCA
HMF	*Ma*AAO	24	0	0	0	99.6	0.4
	*Ma*AAO	144	0	0	0	99	1
	*Ma*AAO + UPO	24	0	0	21	61	18
	*Ma*AAO + UPO	144	0	0	0	0	100
DFF	*Ma*AAO	24	-	0	0	99.6	0.4
	*Ma*AAO	144	-	0	0	99	1
HMFCA^a^	*Ma*AAO	24	-	-	39.4	60	0.4
	*Ma*AAO	144	-	-	0	99.2	0.8
FFCA	*Ma*AAO	24	-	-	-	99	1
	*Ma*AAO	144	-	-	-	60	40

^a^Reaction was conducted in 100 mM sodium acetate buffer, pH 5.0

## Discussion

The implementation of AAOs as biocatalysts for the production of precursors for bio-based polymers, flavors, fragrances, or pharmaceutical compounds is hampered by the low expression level of most of these enzymes and the limited number of AAOs described and characterized so far. The latter one might also be caused by the limited availability of these enzymes. For instance, heterologous expression of *P. eryngii* AAO (*Pe*AAO) in *P. pastoris* required directed evolution of this enzyme and eventually yielded 25.5 mg/l of *Pe*AAO variant FX9 in *P. pastoris* (Vina-Gonzalez et al. 2018). Recently, *Pe*AAO2 from *P. eryngii* P34 was heterologously expressed in *P. pastoris* at 315 mg/l (Jankowski et al. 2020). The putative AAO, *Ma*AAO from *M. antarcticus*, was expressed with its native signal peptide for secretion in *P. pastoris* at 750 mg/l, which is one of the highest reported yields of AAOs so far.

**Fig. 7:**
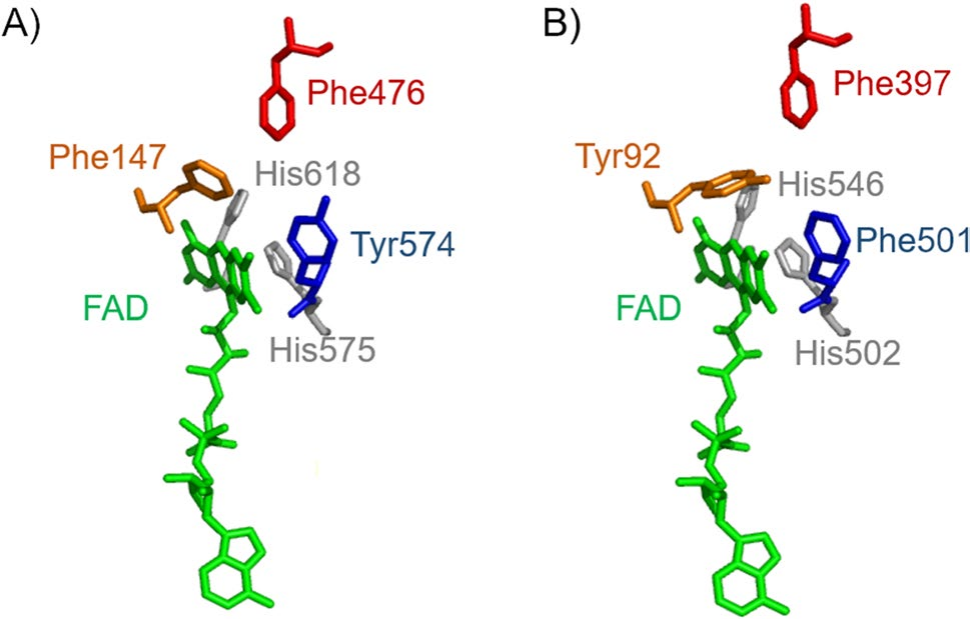
Comparison of the active site of *Ma*AAO (**A**, 3D homology model) and *Pe*AAO (**B**, PDB entry 3FIM) drawn with PyMOL. The FAD molecule (in green), the catalytic histidines (in gray), and the aromatic amino acid residues forming the substrate access channel (in blue, red, and orange) of *Ma*AAO and *Pe*AAO are shown

Identified by BLASTp searches using several known AAO sequences, *Ma*AAO annotated as GMC oxidoreductase was identified. *Ma*AAO contains the two catalytic histidines (His575 and His618 in *Ma*AAO) highly conserved among AAOs as was shown by multiple sequence alignments (Figure S3). As in other AAOs, the substrate access channel is formed by three aromatic amino acid residues (Phe147, Phe476, and Tyr574), that vary among AAOs. *Pe*AAO possesses, for example, Tyr92, Phe397, and Phe501 at the corresponding positions (Fig. 7).

Spectroscopic analysis of native and heat-treated enzyme confirmed that *Ma*AAO contains a non-covalently bound FAD cofactor typically for AAOs. *Ma*AAO has a theoretical molecular mass of 67 kDa (without its predicted N-terminal signal peptide) and possesses six potential *N*-glycosylation sites. The enzyme was expressed with around 11% *N*-glycosylation extent in *P. pastoris*. In comparison, *Pe*AAO variant FX9 with seven potential *N*-glycosylation sites was only poorly glycosylated when expressed in *P. pastoris* (Vina-Gonzalez et al. 2018), while recombinantly expressed *Pe*AAO2 from *P. eryngii* P34 with eight potential *N*-glycosylation sites showed 30% *N*-glycosylation (Jankowski et al. 2020). Viña-Gonzalez and colleagues showed that *N*-glycosylation has a positive effect on thermostability of *Pe*AAO when compared to non-glycosylated *Pe*AAO expressed in *E. coli* (Vina-Gonzalez et al. 2015). Thermostability of *Ma*AAO was quite high with 60% remaining activity after 3 h of incubation at 70 °C while activity of similarly glycosylated *Pe*AAO expressed in *Aspergillus nidulans* dropped to ~ 10% after 40 min incubation at 65 °C (Ruiz-Duenas et al. 2006) and *Mt*AAOx from *Thermothelomyces thermophilus* M77 remained only ~ 10% of its activity after 2 h incubation at 70 °C in the presence of calcium (Kadowaki et al. 2020). Moreover, the *T*_50_ value of *Ma*AAO (74 °C) is the highest of an AAO reported so far and much higher than that of heavily glycosylated *Pe*AAO (58.8 °C) expressed in *S. cerevisiae* (Vina-Gonzalez et al. 2015) or *Pe*AAO2 (62.1 °C) expressed in *P. pastoris* (Jankowski et al. 2020). Glycosylated *Ma*AAO also showed high stability from pH 2 to 9 and is more stable under acidic conditions compared to other AAOs (Jankowski et al. 2020; Vina-Gonzalez et al. 2015). Besides this, *Ma*AAO was quite active and stable in the presence of the ionic liquids (ILs) choline acetate and choline dihydrogen phosphate. ILs are salts that exist in liquid form often below 100 °C. They have gained increasing attention over the last years and become promising reaction media for biocatalytic reactions (Elgharbawy et al. 2020). ILs have not been investigated as cosolvents in AAO-catalyzed reactions so far. However, the positive effect of the bio-based IL choline dihydrogen phosphate on enzyme activity and stability has been already described by Galai and coworkers for *Trametes versicolor* laccase (Galai et al. 2015). The high pH and thermal stability together with its high activity and stability in the presence of ILs and hydrogen peroxide makes this enzyme a promising biocatalyst for application in synthesis of value-added compounds.

The substrate spectrum of *Ma*AAO is quite broad and comprises a large number of benzylic alcohols, aliphatic allylic primary alcohols as well as furan derivates, and heterocyclic alcohols. Oxidation of aldehydes was much lower compared to the corresponding alcohols as described for other AAOs (Ferreira et al. 2010; Serrano et al. 2020). Furthermore, activity of *Ma*AAO towards eugenol, a typical substrate of vanillyl alcohol oxidases, was negligible and no activity towards sugars was found, confirming the classification of *Ma*AAO to AAOs (EC 1.1.3.7).

Activity of *Ma*AAO was generally enhanced towards hydroxy-, methoxy-, or amino-substituted benzylic alcohols as compared to benzyl alcohol while, for example, activity of *Pe*AAO2 from *P. eryngii* P34 towards amino-substituted benzylic alcohols was 5 to 10 times lower as compared to benzyl alcohol (Jankowski et al. 2020). Furthermore, *Ma*AAO oxidized benzylic alcohols substituted with a methoxy group at the *meta-* or *para-*position of the aromatic ring equally well as was shown for some other AAOs like r*Cc*AAO from *Coprinopsis cinerea*, BAO from *Botrytis cinerea*, and AOx from *Aspergillus terreus* (Urlacher and Koschorreck 2021). Other AAOs, like the well-studied *Pe*AAO, showed higher activity towards benzylic alcohols with methoxy-substitution in *para*-position than in *meta*-position.

*Ma*AAO accepts both, phenolic and non-phenolic substrates, while, e.g., vanillyl alcohol oxidase oxidizes 4-hydroxybenzylic compounds (Ewing et al. 2020). Phenolic vanillyl alcohol and non-phenolic veratryl alcohol were even oxidized at similar turnover numbers by *Ma*AAO as was shown for some other AAOs (Goetghebeur et al. 1992; Romero et al. 2009; Tamaru et al. 2018).

*K*_M_ and *k*_cat_ values of *Ma*AAO for the investigated substrates were quite similar to r*Cc*AAO from *C. cinerea* (Tamaru et al. 2018), but lower as compared to those of *Pe*AAO, *Pe*AAO2, and BAO. Concerning the catalytic constants and the substrate specificity, *Ma*AAO resembles more r*Cc*AAO than other AAOs, although the amino acid sequence identity of both AAOs is only 31%. Enzyme inhibition at substrate excess has, however, not been reported for r*Cc*AAO as was observed for *Ma*AAO with some of the investigated substrates. Only for *Um*AAO substrate inhibition for some compounds with a very low *K*_M_ value has been described (Couturier et al. 2016).

*Ma*AAO was also active towards cumic alcohol. The oxidation product, cuminaldehyde, is the major component of essential oils obtained from cumin seeds and showed antimicrobial and anti-biofilm effects against *Staphylococcus aureus* and *E*. *coli* (Monteiro-Neto et al. 2020). Oxidation of cumic alcohol to cuminaldehyde was recently described for *Pe*AAO2 with a slightly lower relative activity (149%) than *Ma*AAO (167%) (Jankowski et al. 2020). Piperonyl alcohol was the best substrate among the benzylic alcohols tested for *Ma*AAO with 2.5-times faster conversion compared to benzyl alcohol. The oxidation product piperonal, also known as heliotropin, is used in the fragrance and flavor industry due to its vanilla-like aroma and serves as intermediate for the production of insecticides and pharmaceuticals (Brum et al. 2019; Santos et al. 2004; Wang et al. 2019). Surprisingly, the non-aromatic primary alcohol (*S*)-perillyl alcohol was accepted as substrate by *Ma*AAO and oxidized almost two times better than benzyl alcohol. The oxidation product of the reaction, perillaldehyde, is used as flavoring ingredient to add spiciness to foods and shows several health-promoting properties like antioxidative, antibacterial, anti-inflammatory, and antiallergic effects (Ahmed 2018; Fuyuno et al. 2018; Uemura et al. 2018). Oxidation of this monocyclic monoterpene by an AAO has to the best of our knowledge not been described so far and further expands the substrate scope of AAOs.

The oxidation of HMF and its derivatives makes *Ma*AAO quite interesting for application as biocatalyst in enzymatic synthesis of FDCA. While the chemical route to FDCA requires high temperature and pressure, organic solvents, and metal catalysts (Sajid et al. 2018), some enzymes were shown to catalyze one or more of the individual reaction steps under mild reaction conditions without cosolvents (Carro et al. 2015; Daou et al. 2019; Dijkman and Fraaije 2014; Karich et al. 2018; Mathieu et al. 2020; Vinambres et al. 2020). For example, 5-hydroxymethylfurfural oxidase (HMFO) from *Methylovorus* sp. strain MP688 was shown to oxidize HMF to FDCA via DFF and FFCA, but conversion was not complete (Dijkman and Fraaije 2014). *Pe*AAO oxidized HMF predominantly to FFCA due to hydrogen peroxide formation inhibiting further oxidation of FFCA to FDCA (Serrano et al. 2019a). Among the tested furan derivatives, *Ma*AAO showed highest activity towards HMF and the catalytic efficiency of *Ma*AAO for HMF is in the same range or even higher compared to other AAOs and HMF-oxidizing enzymes (Carro et al. 2015; Daou et al. 2019; Dijkman and Fraaije 2014; Mathieu et al. 2020; Vinambres et al. 2020). However, although FFCA was slowly oxidized to FDCA by *Ma*AAO, only trace amounts of FDCA were detected when starting from HMF. Remarkably, *Ma*AAO was able to completely oxidize HMFCA to FFCA which has not been shown for any other AAO so far. Conversion of HMFCA to FFCA enables the use of a two-enzyme system for synthesis of FDCA, employing UPO for FDCA production from FFCA, while *Ma*AAO supplies UPO with hydrogen peroxide and re-introduces HMFCA, formed by UPO from HMF, back into the reaction as FFCA. This simplifies AAO/UPO-reaction cascades for the production of FDCA relying on a third enzyme like galactose oxidase to oxidize UPO-formed HMFCA to FFCA (Karich et al. 2018). The two-enzyme system enabled complete conversion of HMF to FDCA and optimization of the reaction conditions to improve the conversion rate is under investigation yet. The construction of *Ma*AAO/UPO fusion enzymes might further enhance FDCA production and lead to promising biocatalysts for the synthesis of bioplastic precursors, pharmaceuticals, and other value-added compounds as was recently shown by the use of an evolved peroxygenase-AAO fusion for the synthesis of dextrorphan (de Santos et al. 2020).

In summary, *Ma*AAO from *M. antarcticus* is a new AAO with promising properties that is expressed at high levels in *P. pastoris*. Its broad substrate spectrum and high thermal as well as pH stability render this enzyme a highly attractive biocatalyst for biotechnological applications. Oxidation products of *Ma*AAO-catalyzed reactions can be applied, for example, as precursors for bioplastics, flavors, fragrances, and intermediates for pharmaceuticals. Implementation of AAO-mediated reactions in biotechnological processes will thus contribute to the development of environmentally friendly production routes of value-added compounds.

## Supplementary Information

Below is the link to the electronic supplementary material.

Supplementary file1 (PDF 341 KB)

## Data Availability

The data that support the findings of this study are available from the corresponding author upon reasonable request.
