# Supplementary material for: Characterization of a thermotolerant aryl-alcohol oxidase from Moesziomyces antarcticus oxidizing 5-hydroxymethyl-2-furancarboxylic acid
Source: Appl Microbiol Biotechnol. 2021 Oct 13;105(21-22):8313–27. doi: 10.1007/s00253-021-11557-8 (PMC8557139; doi:10.1007/s00253-021-11557-8)
Supplement: Supplementary file 1 — Supplementary file1 (PDF 341 KB) [file 253_2021_11557_MOESM1_ESM.pdf]

SUPPLEMENTARY MATERIAL

Characterization of a thermotolerant aryl-alcohol oxidase from *Moesziomyces antarcticus* oxidizing 5-hydroxymethyl-2-furancarboxylic acid

Alessa Lappe [1], Nina Jankowski [1], Annemie Albrecht [1], Katja Koschorreck [1,*]

[1] Institute of Biochemistry, Heinrich-Heine University Düsseldorf, Universitätsstraße 1, 40225 Düsseldorf, Germany

[*] Corresponding Author:

Katja Koschorreck

E-Mail: Katja.Koschorreck@hhu.de

ORCID ID 0000-0001-9689-9863

SUPPLEMENTARY MATERIAL AND METHODS

Expression and purification of UPO

The gene encoding for the unspecific peroxygenase *Aae*UPO form *Agrocybe aegerita* (GenBank accession number FM872458.1) containing additional mutations of *Aae*UPO variant PaDa-I (Molina-Espeja et al. 2014) was synthesized and ligated into the pPICZB vector by BioCat GmbH (Heidelberg, Germany) using restriction sites EcoRI and NotI. *P. pastoris* X-33 cells were transformed with MssI linearized pPICZB_*upo* by electroporation. Recombinant cells were selected on yeast extract peptone dextrose sorbitol agar plates (YPDS; 10 g/l yeast extract, 20 g/l peptone, 20 g/l glucose, 1 M sorbitol, 20 g/l agar) supplemented with 100 µg/ml of Zeocin™ (InvivoGen, San Diego, USA). Cells were grown for four days at 30 °C. For expression of UPO in shaking flasks *P. pastoris* transformants were grown in 10 ml buffered complex glycerol medium (BMGY; 10 g/l yeast extract, 20 g/l peptone, 100 mM potassium phosphate buffer pH 6, 13.4 g/l yeast nitrogen base without amino acids, 0.4 mg/l biotin, 10

g/l glycerol) at 30 °C and 200 rpm overnight. Precultures were used for inoculation of 10 ml buffered methanol minimal medium (BMM; 13.4 g/l yeast nitrogen base without amino acids, 100 mM potassium phosphate buffer pH 6, 0.4 mg/l biotin, 0.5 % (v/v) methanol) to an optical density at 600 nm (OD_{600}) of 0.5 and 10 µM hemin was added. Cells were grown for 3 days at 25 °C and 200 rpm. Methanol (0.5 % (v/v)) was added daily. Volumetric activity of the cell-free supernatants was measured daily towards 2,2'-azino-bis(3-ethylbenzthiazoline-6-sulfonic acid) (ABTS) at a concentration of 0.5 mM in 100 mM sodium citrate buffer pH 4.4 with 1.2 mM H_2O_2. Oxidation of ABTS was followed at 420 nm.

Fed-batch fermentation of the most active *P. pastoris* transformant was conducted in a 7.5 l bioreactor (Infors, Bottmingen, Switzerland) as described earlier (Jankowski et al. 2020). Additionally, hemin was added to a final concentration of 10 µM at the time of induction. Samples were taken daily to monitor OD_{600} and volumetric activity towards ABTS. After eight days of cultivation the fermentation broth was harvested by centrifugation for 15 min at 10,000 *g* and 4 °C. The cell-free supernatant was concentrated and rebuffered in 50 mM potassium phosphate buffer pH 7.0 by tangential flow filtration with cut-off membranes of 10 kDa (Pall, Port Washington, USA). Two ml of concentrated supernatant was loaded onto a Butyl Sepharose HP medium (20 ml, GE Healthcare, Chicago, USA) connected to an ÄKTApurifier FPLC-system (GE Healthcare, Chicago, USA). The column, equilibrated with 50 mM potassium phosphate buffer pH 7.0 with 1.5 M ammonium sulfate (eluent B), was washed with four column volumes (CV) with eluent B at a flow rate of 2 ml/min. Proteins were eluted with 75 % of eluent B (eluent A was 50 mM potassium phosphate buffer pH 7.0) for two CV, followed by 35 % of eluent B for two CV to elute UPO. Fractions with a strong absorbance at 418 nm were pooled, concentrated and rebuffered in 50 mM potassium phosphate buffer pH 7.0. Purified UPO was stored at 4 °C.

SUPPLEMENTARY RESULTS

TABLE S1 Kinetic constants K_{M}, k_{cat}, k_{cat}/K_{M} and K_{IU} of *Ma*AAO

Substrate	K_M [µM]	k_{cat} [s^{-1}]	k_{cat}/K_M [mM^{-1} s^{-1}]	K_{IU} [mM]
**3-Aminobenzyl alcohol**	1.74 ± 0.24	6.4	3690	3.27 ± 0.98
***m*-Anisy alcohol**	4.43 ± 2.65	12.3	2769	2.09 ± 0.33
***p*-Anisyl alcohol**	3.54 ± 0.66	10.2	2884	4.28 ± 0.62
**Benzyl alcohol**	< 15.0	11.2	749	1.39 ± 0.35

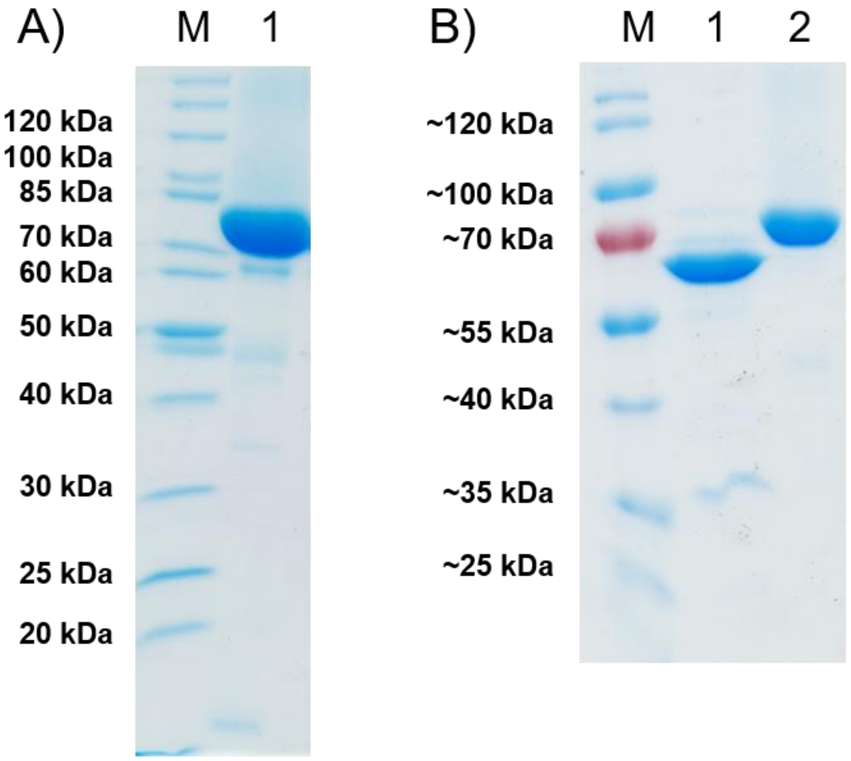

Figure S1. SDS-PAGE analysis of purified *Ma*AAO (lane 1 in A and lane 2 in B) and *Ma*AAO after PNGase F treatment (lane 1 in B). 5 µg of sample was loaded onto the gel. M = Protein ladder.

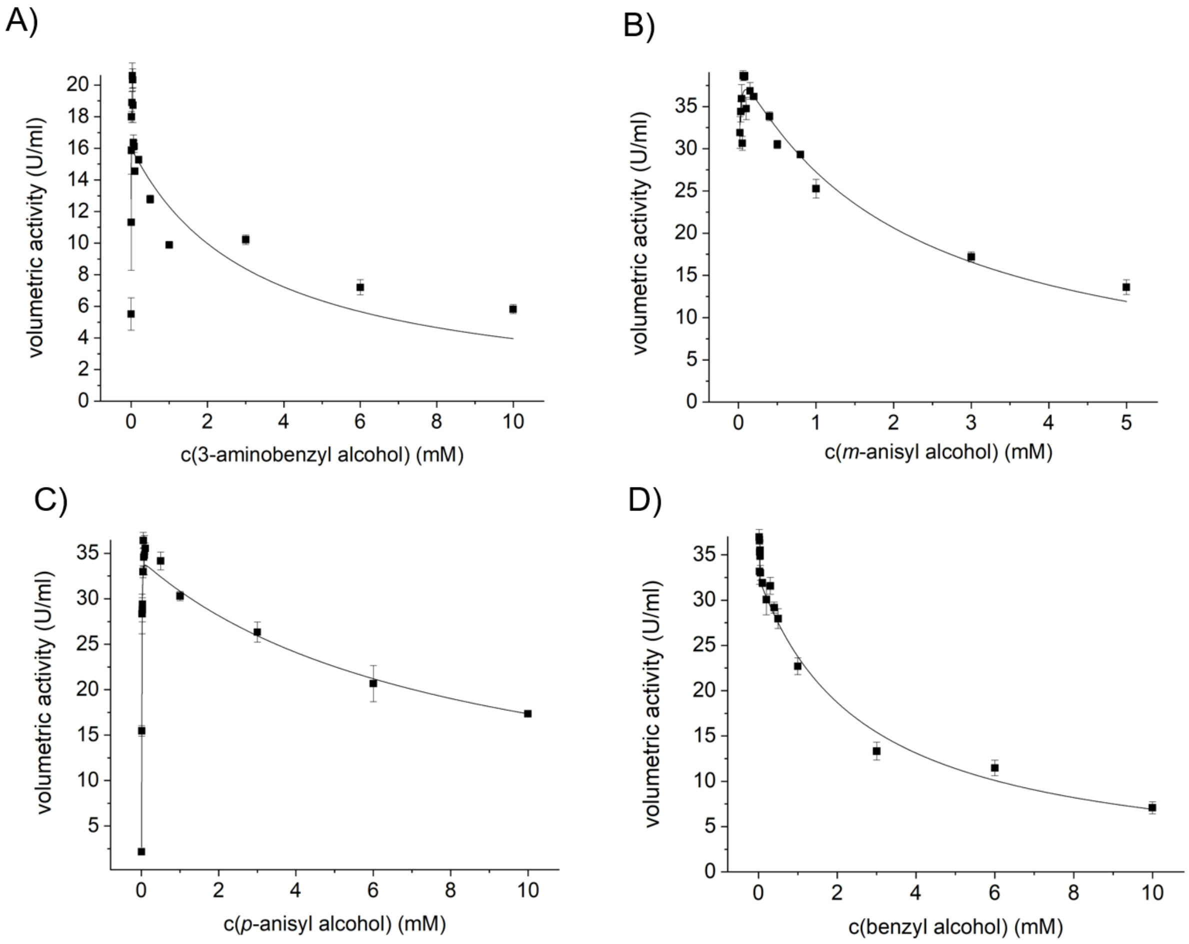

Figure S2. Kinetic analysis of *Ma*AAO for 3-aminobenzyl alcohol (A), *m*-anisyl alcohol (B), *p*-anisyl alcohol (C) and benzyl alcohol (D).

```
AOx           ------------------------------------------------------------     0
MtAAOx        -----------------------------MGF----LAATLVSCAALASAASIPRPHAKR     27
MaAAO         MKATTIIAAAALAGSVAATPVAWTKVSPRSEMAARMAENSHLASRAI-TNDA--------     51
UmAAO         MKTTTLVAAATLAGAVAANPIAWSKVSPRSEFAARMAENSHIAARSI-SSDA--------     51
CcAAO         ---------------------------------------------ALLTDPS--------     7
PeAAO         -----------------------------MSFGA-LRQLLLIACLALPSLAA--------     22
PsAAO         -----------------------------MSFSA-LRQLLFIACLALPSLAA--------     22

AOx           --MTIPDEVDIIICGGGSSGCVPAGRLANLDPSLSVLLIEAGEDNLNNPWVYRPGIYPRN     58
MtAAOx        QVSQLRDDYDFVIVGGGTSGLTVADRLTEAFPAKNVLVIEYGDVHYAPGTFDPPTDWITP     87
MaAAO         -AKFVSKQYDYVVVGAGTAGLALAARLSENGK-YKVGVLEAGGSGYGVGIIDTP-----G     104
UmAAO         -AKFSSKQYDYLVVGAGTAGLAVAARLSESGK-YKVGVLEAGGNGFGVGIIDTP-----G     104
CcAAO         -QLRGDRTYDYVIVGAGNAGNVIAERISAGPHPKSVLVLEAGVSDEGVLAAQVPFLGPTL     66
PeAAO         -TNLPTADFDYVVVGAGNAGNVVAARLTEDPD-VSVLVLEAGVSDENVLGAEAPLLAPGL     80
PsAAO         -ANLPTADFDYIVVGAGNAGNVVAARLTEDPN-VSVLVLEAGVSDENVVGAEAPLLAPGL     80
                       * :: *.*.:* . * *::      .* ::* *           *

AOx           M---KLDSKTASFYYSRPSEHLDGRRAIVPCANILGGGSSINFMMYTRASASDYDDFQ--     113
MtAAOx        QP----DAPPSWSFNSLPNPDMANTTAFVLAGQVVGGSSAVNGMFFDRASRHDYDAWTAV     143
MaAAO         QFGADLGTQYDWNYTTVANPANGVPSSGWPRGRVLGGSSALNFLVWDRSSRYEIDAWE-Q     163
UmAAO         QFGADLGTIYDWNYTTV--PQNGVPAVGWPRGKVLGGSSALNFLVWDRSSRHEIDAWE-Q     161
CcAAO         TPGTFRRTPFDWNYTVAPQEGLDGRTFPFPRGKMLGGCSSVNYMVHHFGSSEDYNKLARD     126
PeAAO         VP----NSIFDWNYTTTAQAGYNGRSIAYPRGRMLGGSSSVHYMVMMRGSTEDFDRYAAV     136
PsAAO         VP----NSIFDWNYTTTAQAGYNGRSIAYPRGRMLGGSSSVHYMVMMRGSIEDFDRYAAV     136
                     :     :                 ..::** *::: :.   .*  : :

AOx           --------AEGWKTKDLVPLMRKHETYQRACNNREL----------HGFD-GPIKVSFGN     154
MtAAOx        GGSGFEQSSHKWDWEGLFPFFQKSVTFTEPPADIVQKYHYTWDLSAYGNGSTPIYSSYPV     203
MaAAO         LG------NPGWNWNNLYKAMKKSERFHAPSQENADLLGVKPVASDYGSS-GPIQVAFPN     216
UmAAO         LG------NPGWNWNNLYSAMKKSEKFHAPSQENADLLGVKPVASDYGSS-GPIQVAFPN     214
CcAAO         SG------DNGWSWSSIKKYIFKHEKIVPPADNSDTDGKF--LPQFHGTG-GTVSVSLPG     177
PeAAO         TG------DEGWNWDNIQQFVRKNEMVVPPADNHNTSGEF--IPAVHGTN-GSVSISLPG     187
PsAAO         TG------DDGWNWDNIQQFVRKNEMVVPPADNHNTSGEF--IPAVHGTN-GSVSISLPG     187
                         *. ..:   . *         :             :* .   :  :

AOx           YTYPIMRDFLRAAESQDIPITDDLQDLKTGHGAEHWLKWI----NRDTGRRSDAAHAYVH     210
MtAAOx        FQWADQPL----LNQAWQEMGINP-VTECAGGDKEGVCWVPASQHPVTARRSHAGLGHYA     258
MaAAO         YISQQVRRWIPALLELGIPKNDQP-L----AGENVGVSQQPSDINPTNYTRSYSAPAYLF     271
UmAAO         YISQQVRRWIPALSELGIPKNDQP-L----AGQNVGVSQQPSNINPSNYTRSYSAPAYLF     269
CcAAO         NSQSIDAKVIATTDEL-PEFPFNP-DQGHGNGQVLGMGWTQN--SIGEGARSSSSTTYLK     233
PeAAO         FPTPLDDRVLATTQEQSEEFFFNP-DMG--TGHPLGISWSIA--SVGNGQRSSSSTAYLR     242
PsAAO         FPTPLDDRVLATTQEQSEEFFFNP-DMG--TGHPLGISWSIA--SVGNGQRSSSSTAYLR     242
                            .       :        *    :             ** :.  :

AOx           STRAKQSNLHLKCNTKVDKVIIENGRAVGVATVPSKPL-DGHDPPRKIFRARKQIIISSG     269
MtAAOx        DVL-PRANYDLLVQHQVVRVVFPNGPSHGPPLVEARS---LADNHLFNVTVKGEVIISAG     314
MaAAO         PNQ-ARSNLDVLTNALASKVNFDS-S-CGELWAKSVTFT--NGGKSYTVNATKEVIISAG     326
UmAAO         PNQ-ARPNLDVLTDALVSKVNFDI-E-CGELSANGVTFI--SNGQTYTVNATKEVILSGG     324
CcAAO         EAL-KRPHVDVLINAHVTKLVTTRKK-RGRPVFDKVQFASGPGAPVTTVTARREIILSAG     291
PeAAO         PAQ-SRPNLSVLINAQVTKLVNSGTT-NGLPAFRCVEYAEQEGAPTTTVCAKKEVVLSAG     300
PsAAO         PAQ-SRPNLSVLINAQVTKLVNSGTT-NGLPAFRCVEYAEREGAPTTTVCAKKEVVLSAG     300
                   : :  :  :  . ::        *             .     . .  ::::*.*

AOx           TLSSPLILQRSGIGDPEKLRAAGIRPLMNLPGVGRNFQDHYLTFSVFRAKPDVES---FD     326
MtAAOx        ALHTPTVLQRSGIGPASFLDDAGIPVTLDLPGVGANLQDHCGPPVTWNYTEPYTGFFPLP     374
MaAAO         TVNTPQLLELSGIGSKDVLGKAGVKVLYENANVGENLQDHTYSATVYNLKSGFKT----L     382
UmAAO         TVNTPQILELSGIGSKDVLSKAGVKVLYENANVGENLQDHTYSATVYKLKPGFPT----L     380
CcAAO         AFGTPQILLLSGIGPKTDLDVLGIPTVIHNPSVGQNLSDHVLLPNIFNVRGQDTL----D     347
PeAAO         SVGTPILLQLSGIGDENDLSSVGIDTIVNNPSVGRNLSDHLLLPAAFFVNSNQTF----D     356
PsAAO         SVGTPILLQLSGIGDQSDLSAVGIDTIVNNPSVGRNLSDHLLLPATFFVNNNQSF----D     356
              :. :* :*  ****    *   *:    .  .** *:.**      :

AOx           DFVRGDPEVQKKVFDEWNLK-GTGPLATNGIDAGV----------------KIRPTEK--     367
MtAAOx        SEMVNNATFKAEAITGFDEVPARGPYTLAGGNNAIFVSLPHLTADYGAITAKIRAMVADG     434
MaAAO         DSLRSDSTFAAEQLAAYKAN-QTSIFTETV-PSISYVSLARVVGAD---RA--KAMIN--     433
UmAAO         DSLRSNTTFAAEQAAAYKAN-QSSILTETV-PSISYVSLARVVGDK---RA--KAMIA--     431
CcAAO         QIIRGDPNVVPGVLDQWTTS-RSGP------------------------------LAN--     374
PeAAO         NIFRDSSE-FNVDLDQWTNT-RTGP------------------------------LTA--     382
PsAAO         NLFRDSSE-FNADLDQWTNT-RTGP------------------------------LTA--     382
              . . ..          :      .
```

```
AOx          ELEEM---KKWPTPEFVDGWET-------YFKN------KPDKPVMHYSVIAGWFGDHML    411
MtAAOx       TAASYLAADVRTIPGMVAGYEAQ----LLVLADLLD---NPEAPSLET----PWAT----    479
MaAAO        EVTQYVQ-------SSRAPYKATLNKQLDFLNNYPD-----KVGQMELIGIDGYFAGTGA    481
UmAAO        EVAKYVG-------ASRAPYKATLKKQLDFLIQYPD-----KIGQMELIGIDGYFAGTGA    479
CcAAO        GVTNNLG-------FFRLPANSS------IFNSVSDPATGPTASHWEMIVINFYLNPFGP    421
PeAAO        LIANHLA-------WLRLPSNSS------IFQTFPDPAAGPNSAHWETIFSNQWFHPAI-    428
PsAAO        LIANHLA-------WLRLPSNSS------IFQSVPDPAAGPNSAHWETIFSNQWFHPAL-    428
                .                ::        :               .      :

AOx          --MPPGKFFTMFHFLEYPFSRGFTHVKSADPYGNPDFDAGFMNDKRDMAAMVWGYIKSRE    469
MtAAOx       --SEAPQTSSVLAFLLHPLSRGSVRLNLSDPLAQPVLDYRSGSNPVDIDLHLAHVRFLRG    537
MaAAO        PKPTE-TYFTILAANQHLFSRGNVHIQSSDPTKYPLIDPKYFSVPFDTELSTAGTAYTRK    540
UmAAO        PKPDE-TYFTILAANQHLFSRGNIHITSNDATKYPSIDAKYFDVPFDLEISTAGTNYTRK    538
CcAAO        PIPPAGTFMTLISALISPTSRGFVRLASADPFTAPIIDPKFLTTQFDIFALREAVRATKR    481
PeAAO        PRPDTGSFMSVTNALISPVARGDIKLATSNPFDKPLINPQYLSTEFDIFTMIQAVKSNLR    488
PsAAO        PRPDTGNFMSVTNALIAPVARGDIKLATSNPFDKPLINPQYLSTEFDIFTMIQAVKSNLR    488
                      ::        :**  ::   :    * ::        *

AOx          TARRMSSYAGEVTAMHPHFAYDSPARAFDLDLETTKAYAGPNHITAGIQHGSWSHPLEKG    529
MtAAOx       -------------------------------LLDTPTMQARG-------ALETAP-----    554
MaAAO        -------------------------------VGLSKAYSDMV-------VGEYWP-----    557
UmAAO        -------------------------------IGLGKTYSDMV-------DSEYWP-----    555
CcAAO        -------------------------------FVTASVWNDYV-------ISPWGG-----    498
PeAAO        -------------------------------FLSGQAWADFV-------IRPFDP-----    505
PsAAO        -------------------------------FLSGQAWADFV-------IRPFDA-----    505
                                            .    .

AOx          NPSLETHLNSHRQDTRNELQYSNEDIKHIEKWVQRHVE-TTWHSLGTCSMAPREGNSLTK    588
MtAAOx       ---------------GSAVA---DSDEALGEYVRSHSTLSFMHPCCTAAMLPEDR-----    591
MaAAO        ------------------------GNVDLQNYTKTTSV-TEYHPIGTASMLPRNQ-----    587
UmAAO        ------------------------GNVDIQEYTKTTSV-TEYHPIGTASMLPRKQ-----    585
CcAAO        ---------------LAQ-----TSDEGIDAYVRQQST-TVYHPVGTAAISPRGA-----    532
PeAAO        ---------------RLRDP---TDDAAIESYIRDNAN-TIFHPVGTASMSPRGA-----    541
PsAAO        ---------------RLSDP---TNDAAIESYIRDNAN-TIFHPVGTASMSPRGA-----    541
                                     .   :  : :     :  *   *.:: *.

AOx          HGGVVDERLNVHGVEGLKVCDLSICPDNVGCNTFSTALLIGEKCAMLVAEDLGYSGAALE    648
MtAAOx       -GGVVGPDLKVHGAEGLRVVDMSVMPLLPGAHLSATAYAVGEKAADIIIQEWMDKEQ---    647
MaAAO        -GGVVDPSLRVYGTTNLRVVDASIMPLHVAAHIQATIYGVAEYAASIIKSQA--------    638
UmAAO        -GGVVDPSLRVYGTSNLRVVDASIIPLHVAAHIQATIYGVAEYAAKIIKSQA--------    636
CcAAO        NYGVVDPDLKLKGAEGVRIADASVWPFLPNAHTQGPVYLLAERAADLILGRA--------    584
PeAAO        SWGVVDPDLKVKGVDGLRIVDGSILPFAPNAHTQGPIYLVGKQGADLIKADQ--------    593
PsAAO        SWGVVDPDLKVKGVDGLRIVDGSILPFAPNAHTQGPIYLVGERGADLIKADQ--------    593
               ***.  *.: *. .::: * *: *    .:  .    :.:  * ::

AOx          MKVPTYHAPGEFTGLARL    666
MtAAOx       ------------------    647
MaAAO        ------------------    638
UmAAO        ------------------    636
CcAAO        ------------------    584
PeAAO        ------------------    593
PsAAO        ------------------    593
```

Figure S3. Sequence alignment of *Ma*AAO and other AAOs. The catalytic histidines are highlighted in blue, the aromatic amino acid residues forming the substrate access channel are highlighted in brown. AOx from *A. terreus* (GenBank accession number AFP17823.1), *Mt*AAOx from *T. thermophilus* (GenBank accession number AEO55678.1), *Ma*AAO from *M. antarcticus* (NCBI reference sequence XP_014653549.1), *Um*AAO from *U. maydis* (GenBank accession number KIS68002.1), *Cc*AAO from

C. cinerea (GenBank accession number BBC20609.1), *Pe*AAO from *P. eryngii* (GenBank accession number AAC72747.1) and *Ps*AAO from *P. sapidus* (GenBank accession number AMW87253.1).

SUPPLEMENTAL REFERENCES

Jankowski N, Koschorreck K, Urlacher VB (2020) High-level expression of aryl-alcohol oxidase 2 from *Pleurotus eryngii* in *Pichia pastoris* for production of fragrances and bioactive precursors. Appl Microbiol Biotechnol 104(21):9205-9218. doi:10.1007/s00253-020-10878-4

Molina-Espeja P, Garcia-Ruiz E, Gonzalez-Perez D, Ullrich R, Hofrichter M, Alcalde M (2014) Directed evolution of unspecific peroxygenase from *Agrocybe aegerita*. Appl Environ Microbiol 80(11):3496-507. doi:10.1128/AEM.00490-14
